# Updated cancer risks in lynch syndrome

**DOI:** 10.1007/s10689-026-00598-5

**Published:** 2026-10-20

**Authors:** Christine M. Drogan, Sonia S. Kupfer

**Affiliations:** 1 Department of Medicine, Section of Gastroenterology, Hepatology and Nutrition, University of Chicago, Chicago, IL USA; 2 Center for Clinical Genetics and Genomics, University of Chicago, IL Chicago, United States of America

**Keywords:** Lynch syndrome, Cancer, Cumulative risks, Relative risks

## Abstract

Lynch syndrome, one of the most common hereditary cancer predisposition syndromes, is caused by pathogenic variants in the mismatch repair genes (*MLH1, MSH2, MSH6, PMS2*) and the *EPCAM* gene. Individuals who inherit pathogenic variants in Lynch-associated genes experience increased lifetime risk of colorectal, endometrial, and several other cancer types. Risks of Lynch-associated cancers vary by the affected gene and within each gene, especially for lower penetrance genes. This poses challenges for clinicians to provide patients and families with accurate cancer risk estimates and determine individualized surveillance protocols. Compounding this challenge is that study designs and potential biases could yield different risk estimates. In this review, we present up-to-date Lynch syndrome cancer risk estimates and discuss how study design could impact these risk estimates.

## Introduction

Lynch syndrome (LS), caused by pathogenic variants in the mismatch repair genes (*MLH1, MSH2, MSH6, PMS2*) and *EPCAM,* is one of the most common hereditary cancer syndromes affecting 1 in 279–550 individuals [1, 2]. LS confers increased lifetime risk of several tumor types including, most commonly, colorectal and endometrial cancers as well as extra-colonic cancers (e.g., small intestine, gastric, pancreaticobiliary), urological, ovarian, brain, and skin cancers. Risks of other cancer types such as prostate, breast and other cancer types (e.g., thyroid, sarcoma) in LS are debated, and in some cases, may be gene dependent [3]. Cancer risk estimates vary by the affected LS-associated gene, with pathogenic variants (PVs) in higher penetrance *MLH1* and *MSH2* (both germline and epigenetic silencing by *EPCAM* deletions) genes conferring higher risks of cancers compared to PVs in lower penetrance genes *MSH6* and *PMS2*. Because of these varying risks, guidelines now recommend surveillance according to the affected gene for personalized management [4].

Historically, cancer risks in LS were estimated based on individuals and families who met strict Amsterdam I and II criteria [5, 6]. As such, cancer risks reflected estimates from highly penetrant families with markedly elevated lifetime cancer risks. With the expansion of genetic testing in individuals and families not meeting strict clinical criteria or within the general population, estimates of LS-associated cancer risks are lower. Other factors that may impact cancer risk estimates in LS include age, sex, geographic region, hypomorphic or reduced penetrance variants [7], genetic or familial modifiers [8–10] as well as exposures such as surveillance and surgery, physical activity, body mass index, and aspirin use [11, 12].

Cancer risks in inherited syndromes can be estimated using different study designs such as retrospective or prospective cohort and case–control studies that include clinic-, laboratory- or population-based individuals. Several meta-analyses of individual studies have been undertaken for one or more LS-associated cancers and provide risk estimates based on larger patient numbers with assessment of heterogeneity and potential bias. Risk can be measured as an absolute cumulative risk to a certain age (e.g., penetrance) or as relative risk (e.g., hazard, standardized incidence or odds ratio). Cumulative risk can be evaluated by gene, age and sex for individualized estimates and is often used when counseling an individual patient about lifetime risks and surveillance. Relative risks provide information about the strength of the association of a particular cancer by LS gene. These estimates are subject to biases such ascertainment bias and healthy volunteer bias which could lead to over- and underestimation of cancer risks.

In this review, we provide up-to-date information about cancer risks in LS. Given the large number of studies on LS cancer risks published to date, we focus primarily on meta-analyses and recent studies reporting on large numbers of LS patients. Types of studies and potential biases are discussed to help clinicians understand and interpret cancer risk estimates.

## Study design, potential bias, and risk estimation

Understanding study design and potential biases is important when interpreting cancer risk estimates. An ideal design to determine LS cancer risks would be a large, prospective, longitudinal population-based study with representation across all LS genes and verification of data completeness and quality. Given that an ideal study design is not always feasible, we highlight limitations and biases of various study types that were recently discussed in a published commentary [13].

*Study design and potential bias:* Most published studies on cancer risks in LS are retrospective [14]. These studies include LS patients who were ascertained based on strong personal or family history of cancer and lack representation of lower penetrance genes (e.g., *MSH6, PMS2*). Ascertainment bias, which can lead to overestimated risks, may be corrected by instituting modified segregation analysis or genotype-restricted likelihood, though not all studies apply corrections. Studies that correct for ascertainment bias generally report lower cancer risks than those that do not [14]. Retrospective studies often use non-verified participant-reported family history data that can be inaccurate or misclassified. For example, in LS, some types of cancers are frequently mistaken for other types (ex. endometrial and ovarian), and family history reporting is less accurate the more distantly related from the proband [15]. Surveillance and surgeries among LS patients can also bias cancer risk estimates.

Larger patient populations can be studied retrospectively using population- or laboratory-based cohorts. Population-based studies may underestimate cancer risks, as there may be a bias towards healthy volunteers without a previously known PV. LS has been found to be less common in population-based studies than previous estimates (1 in 310–550 compared to 1 in 279) [1, 2, 16–18]. In population studies, there tend to be more PVs in the low penetrance genes *MSH6* and *PMS2* compared to *MLH1* and *MSH2* [2, 16–18]. Laboratory-based cohorts can also provide cancer risk estimates on many individuals with proven PVs in LS-related genes. These cohorts can be biased due to ascertainment for individuals undergoing genetic testing as well as incomplete personal and family history obtained from test requisition forms.

Prospective studies are comprised of patients ascertained based on personal or family history and may also overestimate risk if not corrected [13]. In contrast to retrospective studies, prospective studies can be more accurate because cancer diagnoses and other relevant information are collected in real time and are verified. However, data in prospective studies can be incomplete or misclassified, and subjects can be lost to follow up. Surveillance and surgeries can similarly impact penetrance estimates. The Prospective LS Database (PLSD) is the largest prospective study to report on LS cancer risks to date [19, 20].

Meta-analyses of cancer risks in LS provide synthesized data that overcomes some limitations of individual studies. Advantages include refinement of risk estimates due to larger sample sizes that are more robust and reliable than individual studies. These approaches have more power to evaluate risks by gene, age, and sex as well as geographic location and can also resolve conflicting information between studies. However, meta-analyses can be limited to the quality of available studies, publication bias and heterogeneity across studies. In this review, we focus on results from meta-analyses for LS-related cancer risks wherever feasible.

*Risk estimation:* Cancer risk in inherited syndromes can be expressed as an absolute or relative risk. Absolute risk, also known as penetrance, expresses the likelihood that a person with a PV will develop cancer over a specified time (e.g., 5-year risk, cumulative lifetime risk to age 70). Absolute risk is often used to counsel patients with LS and determine when to start surveillance. Relative risk compares cancer in people with a mutation to those without a mutation and provides information about the strength of risk compared to non-carriers. However, relative risk does not provide direct information about the probability that a PV carrier will develop cancer in their lifetime. In this review, we include both absolute and relative risk estimates by cancer types as well as by gene, sex and geographic location when available.

## Colorectal cancer

Colorectal cancer is the most common cancer type in LS and may have distinct histopathological characteristics such as mucinous, lymphocyte-infiltrating, or signet ring features [21]. Synchronous and metachronous cancers are also more common in people with LS. LS colon cancers are also more likely to be right-sided. Most studies do not distinguish between histological type or location of the tumor when describing cumulative or relative risk. While risk of metachronous colorectal cancers and incidence of second cancers have been described [22, 23], the studies discussed below report on first colorectal cancer risk.

*Cumulative risks:* Several meta-analyses report on cumulative colorectal cancer risks in LS (Table 1). A recent meta-analysis by Campoy et al. stratified cancer risk by study design [14]. Most included studies were retrospective (n=31) with one population-based study (i.e., UK Biobank) [2] and one prospective cohort study (i.e., PLSD) of 6350 participants with 51,646 follow up years under regular colonoscopy surveillance [19]. When comparing the cumulative risks of colorectal cancer between the different types of studies, the retrospective studies that were not corrected for ascertainment bias reported higher risks than those corrected for ascertainment bias, sometimes with non-overlapping confidence intervals. Cumulative risks of colorectal cancer in the prospective study were slightly higher or similar to the population-based study for most genes and sex. The meta-analysis reported lower colorectal cancer risks for *PMS2* and *MSH6* PV compared to *MLH1* and *MSH2* PV carriers, which was consistent across study types.

**Table 1:** Cumulative colorectal cancer risks to age 70 by LS gene from meta-analyses. Risks reported as % (95% confidence interval)

Study, year	Study Type (number of studies)	*MLH1*		*MSH2/EPCAM*		*MSH6*		*PMS2*	
		M	F	M	F	M	F	M	F
Campoy et al., 2025 [14]	Retrospective, corrected for bias (12)	42.2 (36.6–47.9)	33.8 (28.7–39.4)	41.5 (34.5–40.5)	33.0 (26.3–40.5)	16.8 (13.0–21.4)	10.0 (7.5–13.2)	12.4 (9.4–16.2)	9.0 (6.2–12.7)
	Retrospective, not corrected for bias (19)	60.1 (54.9–65.1)	50.6 (46.0–55.2)	25.4 (18.7–33.6)	53.7 (43.5–63.5)	43.1 (25.3–62.8)	22.2 (16.3–29.6)	–	–
	Population-based (1, UK Biobank)	53.5 (34.7–74.7)	38.2 (23.3–58.4)	31.0 (14.9–57.3)	32.8 (15.5–60.7)	14.6 (9.1–23.0)	11.0 (6.6–18.1)	3.4 (1.3–9.0)	5.0 (2.5–9.9)
	Prospective (1, PLSD)	52.8 (46.1–59.8)	42.1 (36.2–48.6)	51.0 (42.8–59.7)	39.8 (33.5–46.7)	13.5 (7.1–24.8)	17.3 (11.2–26.5)	10.5 (2.7–36.0)	8.5 (2.1–31.5)
Wang et al., 2020 [25]	Meta-analysis (10*)	43.9 (39.6–46.6)	37.3 (32.2–40.2)	53.9 (49.0–59.6)	36.0 (30.6–41.8)	12.0 (2.4–24.6)	12.3 (3.5–23.3)	–	

*LS* Lynch Syndrome, *M* male, *F* female PLSD, Prospective LS Database *5 studies were also included in Campoy et al

Two previous meta-analyses by Jenkins et al. [24] and Wang et al. [25] were reported in 2015 and 2020, respectively. Jenkins et al. included four studies to calculate colorectal cancer risk in the next 5-years by decade of life [24]. Colorectal cancer risks in individuals with LS PVs were elevated compared to the general population in all age groups (ages 20–29, 30–39, 40–49, 50–59, 60–69 and 70–79). Risks for males and females peaked in the 50 s, with an estimated 14% of males and 8.7% of females with LS expected to be diagnosed with colorectal cancer, compared to 0.62% and 0.42% in the general population (males and females, respectively). This study only included individuals with *MLH1* and *MSH2* PVs. While still elevated over the general population, the risk of colorectal cancer decreased in the 60 s and 70 s. All four studies included in Jenkins et al. were included in subsequent meta-analyses Wang et al. and Campoy et al. [14, 25].

Wang et al. estimated cumulative lifetime risks of colorectal cancer based on data from 10 studies for *MLH1*, *MSH2* and *MSH6* [25]. Generally, colorectal cancer risks were higher in males than females, and higher in *MLH1/MSH2* compared to *MSH6*. Five of the 10 studies included in Wang et al. were also included in the Campoy et al. meta-analysis [14].

*Relative risks:* Table 2 summarizes relative risks of colorectal cancer in LS from population- and laboratory-based cohorts not included in the most recent meta-analysis [14]. Two population-based studies, All of Us and the New York -based Bio*Me* studies reported LS in 0.3% and 0.2% of individuals, respectively [16, 18]. Colorectal cancer was present in 10.9% and 4% of LS carriers, respectively. In a North American based laboratory cohort previously presented at an international conference, all five LS genes were associated with an increased risk of colorectal cancer [26]. Manuscript is pending and will be submitted for publication. Of the 25,408 individuals with colorectal cancer in this cohort, 7.6% had a PV in a LS gene, most commonly in *MLH1* (2.3%) and *MSH2* (2.3%). 13/34 (38%) of patients with *EPCAM* PV had a diagnosis of colorectal cancer [26]. The relative risks of colorectal cancer were lower than the cohort from North America included in a retrospective study from the International Mismatch Repair Consortium [9].

**Table 2:** Relative colorectal cancer risks in LS from population- and laboratory-based cohorts. Reported as relative risks (95% confidence interval)

Study, year	Age	Geographic location	All LS Genes	*MLH1*		*MSH2*		*MSH6*		*PMS2*		*EPCAM*
				M	F	M	F	M	F	M	F	
Park et al., 2025*[16]			9.2 (6.6, 12.4)	–		–		–		–		–
Rosenblum et al., 2020* [18]			5.0 (1.3–18.8)	–		–		–		–		–
Win et al. 2021** [9]	At age 40	Europe	–	37.5 (15.7–89.7)	23.4 (9.0–61.0)	27.9 (12.8–60.4)	25.2 (10.3–61.6)	14.8 (4.4–50.2)	3.0 (0.8–11.0)	6.7 (1.7–26.7)	1.1 (0.2–6.6)	–
		North America	–	165.3 (103–266.0)	72.1 (42.0–123.8)	126.0 (84.6–187.0)	81.0 (51.1–128.6)	29.3 (12.0–71.9)	2.6 (0.21–31.3)	9.8 (1.8–53.3)	6.7 (1.7–26.7)	–
		Australasia	–	138.0 (71.0–267.0)	117.0 (59.2–232.0)	156.0 (68.9–351.2)	101.5 (37.0–279.0)	20.5 (6.2–67.8)	3.9 (0.9–16.8)	26.8 (5.7–127.0)	7.06 (1.07–45.66)	–
	At age 60	Europe	–	35.8 (15.0–85.7)	22.3 (8.7–57.3)	18.2 (6.67–49.6)	13.0 (4.1–41.3)	4.3 (1.3–14.3)	3.3 (1.2–9.1)	2.16 (0.73–6.39)	4.1 (1.6–10.2)	–
		North America	–	32.2 (12.5–82.8)	32.9 (15.7–69.1)	18.4 (8.10–42.0)	7.16 (2.9–17.7)	10.2 (3.6–28.7)	7.2 (2.9–17.7)	4.90 (0.88–27.42)	2.0 (0.5–8.7)	–
		Australasia	–	13.3 (3.4–52.9)	15.9 (4.4–57.7)	13.3 (3.4–52.9)	6.17 (1.3–28.6)	2.7 (0.4–16.7)	3.9 (0.9–17.0)	2.2 (0.3–13.5)	2.1 (0.4–10.7)	–
Drogan et al. 2024* [26]	–	–	–	14.4 (12.5–16.5)		8.01 (7.08–9.05)		3.4 (3.0–3.9)		2.2 (1.9–2.5)		4.7 (2.0–11.0)

*LS* Lynch Syndrome, *CI* confidence interval, *M* male, *F* female *odds ratio; **hazard ratio

A large, multinational retrospective study reported colorectal cancer risks by gene, age (40 or 60), sex and continent (Table 2) [9]. Males were generally at higher risks of colorectal cancer. This difference was more prominent when comparing sex differences at age 40. There was also variance in colorectal cancer risk between continents, even though guidelines from these areas are similar in their colonoscopy initiation age and frequency, indicating that other factors beyond mutated gene and sex contribute to cancer risk.

## Endometrial cancer

The most common histological type of endometrial cancer in LS is endometrioid, but clear cell, papillary serous, and malignant mixed mullerian tumors have also been reported [27]. All subtypes are typically grouped together in studies estimating cumulative and relative risks.

*Cumulative risks:* Cumulative risks of endometrial cancer are reported in Table 3. The meta-analysis from Campoy et al. also assessed cumulative endometrial cancer risk in LS noting that the risks of endometrial cancer are similar in *MLH1*, *MSH2* and *MSH6* carriers, and lower in *PMS2* carriers [14]. This difference was consistent across study types. Endometrial cancer was rare (0.9%) in female *MSH6* PV carriers under age 50 [28].

**Table 3:** Cumulative endometrial cancer risks to age 70 by LS gene in a meta-analysis. Reported as % (95% confidence interval)

Study, year	Study Type (number of studies)	*MLH1*	*MSH2/EPCAM*	*MSH6*	*PMS2*
Campoy et al., 2025 [14]	Retrospective, corrected for bias (12)	41.3 (14.3–74.8)	29.2 (18.9–42.2)	22.3 (14.0–33.5)	13.6(8.0–22.2)
	Retrospective, not corrected for bias (19)	32.1 (20.4–46.5)	25.4 (18.7–33.6)	49.8 (29.8–69.9)	–
	Population-based (UK Biobank)	27.7 (15.2–47.2)	23.7 (9.0–54.1)	26.5 (19.2–36.1)	2.6 (1.1–6.3)
	Prospective (PLSD)	35.8 (29.9–42.5)	42.1 (35.0–50.0)	41.4 (32.3–52.0)	12.7 (5.5–27.9)

*LS,* Lynch Syndrome *PLSD* Prospective LS Database

A 2026 meta-analysis of 10 studies reported on gynecologic and breast cancer risks in over 12,000 females with LS [29]. Three of the studies in this meta-analysis were also included in the Campoy et al. study. This study reported prevalence, rather than cumulative risk, of cancer. Prevalence measures the total burden of a particular disease (new and existing cases), while cumulative risk measures the probability that the disease will develop over a certain timeframe (new diagnoses only). Prevalence may be higher than cumulative risk, so caution should be taken when comparing these statistics. The overall prevalence of endometrial cancer in LS carriers was 20%, with *MSH2* (21%) and *MSH6* (18%) having higher prevalence than *MLH1* (11%) and *PMS2* (8%) [29].

This study also compared odds ratios for endometrial cancer between the different LS genes, rather than comparing the risk conferred by each LS gene to the risk in the general population. They found that *MSH6* carriers had the highest estimated risk of endometrial cancer compared to the other LS genes (1.46 CI 1.02–2.04) [29].

*Relative risks:* Relative risks of endometrial cancer are summarized in Table 4. In population studies, endometrial cancer varied from 9.3 to 18% [16, 18]. In the laboratory-based cohort, endometrial cancer was elevated for all mismatch repair genes [26]. Like other studies, risks were comparable in *MLH1*, *MSH2* and *MSH6* carriers, and lower for *PMS2* carriers. Of the 19,328 cases of endometrial cancer, a PV was detected in 1153 (5.96%). The most common mutated gene was *MSH6* (1.6%).

**Table 4:** Relative endometrial cancer risks in LS from population- and laboratory-based cohorts. Reported as odds ratios (95% confidence interval)

Study, year	All LS Genes	*MLH1*	*MSH2*	*MSH6*	*PMS2*	*EPCAM*
Park et al., 2025 [16]	10.7 (7.1–16.2)	–	–	–	–	
Rosenblum et al., 2020 [18]	30.8 (9.6–99.0)	–	–	–	–	
Drogan et al., 2024 [26]	–	5.0 (4.1–6.1)	6.2 (5.3–7.2)	6.0 (5.3–6.7)	2.1 (1.8–2.5)	0.6 (0.1–4.3)

*LS,* Lynch Syndrome

## Extra-colonic gastrointestinal cancers

Several extracolonic GI cancers have been associated with LS, including gastric, small bowel, pancreas, and biliary, summarized in Tables 5 and 6.

**Table 5:** Cumulative extracolonic gastrointestinal cancer risks to age 70 by LS gene. Reported as % (95% confidence interval)

Cancer Type	Study, year	Study Type (number of studies)	*MLH1*		*MSH2/EPCAM*		*MSH6*		*PMS2*	
			M	F	M	F	M	F	M	F
Gastric	Campoy et al., 2025 [14]	Retrospective, corrected for bias (12)	16.5 (6.8–35.1)	7.2 (2.8–17.5)	1.3 (0.2–8.5)	2.3 (0.1–49.7)	–	–	–	–
		Retrospective, not corrected for bias (19)	5.7 (2.7–11.6)	5.7 (2.7–11.6)	6.5 (4.8–8.9)	6.5 (4.8–8.9)	10.4 (3.4–17.3)	10.4 (3.4–17.3)	–	–
		Prospective (1, PLSD)	5.5 (3.3–9.3)	2.9 (1.5–5.6)	5.0 (2.9–8.5)	3.1 (1.7–5.9)	0.7 (0.1–4.9)	0.7 (0.1–4.7)	2.7 (0.4–17.5)	–
Biliary	Campoy et al., 2025 [14]	Retrospective, corrected for bias (12)	1.9 (0.0–15.0)	0.0 (0.0–0.2)	1.9 (0.0–15.0)	0.0 (0.0–0.2)	–	–	–	–
		Retrospective, not corrected for bias (19)	3.8 (2.7–5.3)	3.8 (2.7–5.3)	1.3 (0.1–11.9)	1.3 (0.1–11.9)	–	–	–	–
		Prospective (1, PLSD)	4.0 (2.2–7.3)	1.5 (0.7–3.3)	2.3 (0.9–5.7)	0.8 (0.3–2.4)	–	–	–	–
Small bowel	Campoy et al., 2025 [14]	Retrospective, corrected for bias (12)	0.4 (0.1–3.0)	0.4 (0.1–3.0)	1.1 (0.0–5.0)	1.1 (0.0–5.0)	–	–	–	–
		Retrospective, not corrected for bias (19)	4.0 (2.5–6.3)	4.0 (2.5–6.3)	3.3 (1.0–9.9)	3.3 (1.0–9.9)	–	–	–	–
		Prospective (1, PLSD)	6.6 (4.1–10.3)	3.8 (2.2–6.7)	7.0 (4.3–11.3)	3.7 (2.1–6.4)	1.6 (0.4–6.5)	0.6 (0.1–4.0)	3.3 (0.5–21.3)	2.1 (0.3–14.0)
Pancreas	Campoy et al., 2025 [14]	Retrospective, not corrected for bias (19)	–	–	0.7 (0.0–1.4)	0.7 (0.0–1.4)	–	–	–	–
		Prospective (1, PLSD)	2.2 (0.9–5.1)	2.3 (1.1–4.7)	3.3 (1.5–7.1)	2.7 (1.3–5.8)	–	2.2 (0.7–6.8)	–	–
	Bogdanski et al., 2026^+^ [34]	Retrospective (1)	0.8 (0.3–2.2)		1.7 (0.9–3.4)		0.2 (0.0–1.3)		0 (0.0-0.0)	
Ampullary	Bogdanski et al., 2026 [34]	Retrospective (1)	0.0 (0.0)		0.5 (0.1-1.6)		0.2 (0.0–1.5)		0 (0.0-0.0)	
Distal Bile duct	Bogdanski et al., 2026 [34]	Retrospective (1)	0.3 (0.0–2.3)		0 (0.0-0.0)		0.2 (0.0–1.4)		0 (0.0-0.0)	

*LS* Lynch Syndrome ^+^Includes confirmed and suspected pancreatic ductal adenocarcinoma cases, *PLSD* Prospective LS Database

**Table 6:** Relative extracolonic gastrointestinal cancer risks in LS from population- and laboratory-based cohorts. Reported as relative risk (95% confidence interval)

Cancer Type	Study, year	*MLH1*	*MSH2*	*MSH6*	*PMS2*
Gastric	Drogan et al., 2024 [26]	4.6 (2.5–8.2)	2.6 (1.4–4.7)	0.8 (0.3–2.2)	0.5 (0.2–1.7)
Pancreas	Drogan et al. 2024 [26]	0.93 (0.3–3.2)	3.9 (2.3–6.4)	1.5 (0.9–2.5)	1.1 (0.7–1.8)
	Bogdanski et al., 2026* [34]	1.8 (0.7–4.8)	3.8 (1.9–7.4)	0.4 (0.0-Inf)	0.0 (0.0-0.0)
Ampullary	Bogdanski et al., 2026 [34]	0.0 (0.0-0.0)	16.7 (4.1–67.7)	6.7 (0.0-Inf)	0.0 (0.0-0.0)
Distal bile duct	Bogdanski et al., 2026 [34]	10.0 (0.0-Inf)	0.0 (0.0-0.0)	6.7 (0.0-Inf)	0.0 (0.0-0.0)

*LS* Lynch Syndrome, *Includes confirmed and suspected pancreatic ductal adenocarcinoma cases

*Gastric*. A systematic review and meta-analysis of gastric cancer in LS included 1 prospective (i.e., PLSD [19]) and 12 retrospective studies to compute pooled risk ratios for gastric cancer [30]. In the PLSD, gastric cancer was increased in *MLH1* and *MSH2* PV carriers compared to *MSH6* and *PMS2* [19].Relative risks could not be calculated in *PMS2* due to only 6 reported cases in *PMS2* carriers across all studies included. Males had a higher risk of gastric cancer than females (RR 2.8, 95% CI 2.2–3.6). Having a family history of gastric cancer or a personal history of H pylori infection were also associated with increased gastric cancer risk (RR 3.5, 95% CI 2.0–5.8; and 2.8, 95% CI 1.2–6.8, respectively). The authors did not assess the risk of multiple combined risk factors or report cumulative risks, which can limit the utility of the findings from the meta-analysis [31]. Gastric cancer risk varies widely by geographic area, so this should be taken into consideration when providing risk estimates for higher-risk groups, like those residing in Asia. In the meta-analysis by Campoy et al., cumulative risks of gastric cancer were highest for male *MLH1* carriers. Risks could not be calculated from the ascertainment bias-corrected studies for *MSH6* and *PMS2* PV carriers [14].

Two retrospective cohort studies reported an elevated risk of gastric cancer [32, 33]. When risk was evaluated by sex, males had a higher cumulative risk to age 70 of gastric cancer (6.7%; 95% CI 3.1–10.3) compared to females (2.6%; 95% CI 1.1–4.1) [32]. In population studies, risks of gastric cancer were not individually reported due to low numbers [16, 18]. 39/1,059 (3.68%) patients in the laboratory-based cohort with gastric cancer had LS, most commonly in *MLH1* and *MSH2* [26]. *MSH6* and *PMS2* PVs were not associated with an increased risk of gastric cancer. Gastric cancer risks were similar to the meta-analysis reported above for *MSH2* and *MSH6*, though were higher in the laboratory-based cohort for *MLH1* PVs (RR 1.8 95% CI 1.4–2.3) [30].

*Small Bowel*. Cumulative risk of small bowel cancer was elevated for *MLH1* and *MSH2* in PLSD [19] but could not be calculated from the ascertainment bias-corrected studies for *MSH6* and *PMS2* PV carriers [14].

In a retrospective study, the standardized incidence ratio (SIR) for small bowel cancer was 10.54 (95% CI 5.9–17.35) [33]. When risk was evaluated by sex, males had a higher cumulative risk to age 70 of small bowel cancer (12.0%; 95% CI 5.7–18.2) to females (3.9%; 95% CI 1.2–6.5) [32]. Risk of small bowel cancer could not be calculated in the laboratory-based cohort or population studies due to low power [16, 18, 26].

*Pancreaticobiliary.* A nation-wide Dutch retrospective study calculated the combined cumulative risks of pancreatic, ampullary and distal cholangiocarcinoma in LS [34]. The risks of these cancers by age 75 was similar in *MSH2* and *MLH1* carriers at 3.4% (95% CI 2.0–5.8) and 3.0% (95% CI 1.5–5.8), respectively. The risk of these cancers was only 1% (95% CI 0.3–2.7%) in *MSH6* carriers. No *PMS2* PV carriers (0/233) in this study were diagnosed with these cancer types. Cumulative risk of pancreatic cancer could not be estimated for any gene in the retrospective studies corrected for ascertainment bias in the Campoy et al. meta-analysis [14].

A study of 147 LS families from two academic registries in the US reported that 20% of LS families had at least one diagnosis of pancreatic cancer [35]. 50% of males with pancreatic cancer were diagnosed before age 50, compared to 22% of females. Of note, *PMS2* PV carriers were not included in this study. The cumulative risk of pancreatic cancer (all LS genes) was 1.3% (95% CI 0.31–2.23) by age 50 3.7% (95% CI 1.45–5.88) by age 70 [35]. Another study reported similar findings (SIR 3.09 95% CI 1.69–5.18) [33].

Risks of pancreaticobiliary cancers could not be calculated due to low power in the population-based studies [16, 18]. Pancreatic cancer risk was only associated with *MSH2* PVs in the laboratory-based cohort [26]. In PLSD, the risk of pancreatic cancer was increased in *MLH1* and *MSH2* PV carriers compared to *MSH6* PV carriers, though only risks for female MSH6 carriers was reported in the Campoy et al. meta-analysis [14, 19].

## Ovarian, urological, brain and skin cancers

Some non-GI, non-endometrial cancers have been reported in LS. Ovarian, urothelial, brain and skin cancer are commonly seen in families with LS. Cumulative risks and relative risks are summarized in Tables 7 and 8.

**Table 7:** Cumulative other cancers risks to age 70 by LS gene. Reported as % (95% confidence interval)

Cancer Type	Study, year	Study Type (number of studies)	All LS Genes	*MLH1*		*MSH2*		*MSH6*		*PMS2*	
				M	F	M	F	M	F	M	F
Ovarian	Campoy et al, 2025 [14]	Retrospective, corrected for bias (12)	–	–	13.6 (6.6–25.9)	–	12.1 (5.2–25.9)	–	1.0 (0.0–3.0)	–	–
		Retrospective, not corrected for bias (19)	–	–	5.5 (3.4–8.6)	–	8.4 (6.6–10.6)	–	–	–	–
		Prospective (1, PLSD)	–	–	8.0 (5.3–12.0)	–	13.4 (8.9–20.1)	–	6.3 (2.6–15.2)	–	2.5 (0.4–16.3)
Ureter/ Kidney	Campoy et al, 2025 [14]	Retrospective, corrected for bias (12)	–	0.5 (0.1–3.0)	0.9 (0.1–11.5)	4.5 (1.3–14.6)	5.1 (1.1–20.3)	0.7 (0.0–2.1)	0.7(0.0–2.1)	–	–
		Retrospective, not corrected for bias (19)	–	2.7 (1.9–3.9)	2.0 (1.4–3.0)	9.3 (4.2–19.5)	7.2 (4.8–10.7)	2.2 (1.1–4.6)	2.9 (1.2–4.4)	–	–
		Prospective (PLSD)	–	3.7 (1.9–6.9)	2.2 (1.0–4.6)	13.4 (9.7–18.5)	13.9 (10.2–18.6)	3.3 (1.2–8.7)	3.9 (1.8–8.6)	–	–
Bladder	Campoy et al, 2025 [14]	Retrospective, not corrected for bias (19)	–	5.3 (1.4–18.2)	2.9 (1.3–4.2)	6.9 (2.5–18.1)	3.4 (2.0–5.7)	1.5 (0.7–3.2)	1.7 (0.4–2.9)	–	–
		Prospective (1, PLSD)	–	3.8 (2.1–6.9)	2.7 (1.3–5.5)	7.2 (4.6–11.3)	6.2 (3.9–9.8)	3.0 (1.1–7.9)	2.6 (1.0–6.8)	–	–
Skin (all types)	Adan et al, 2018 [40]	Retrospective (1)	21.1± 4.5%	–		–		–		–	
Brain	Campoy et al, 2025 [14]	Retrospective, not corrected for bias (19)	–	0.7 (0.1–3.9)	0.7 (0.1–3.9)	2.8 (1.1–6.7)	2.8 (1.1–6.7)	–	–	–	–
		Prospective (1, PLSD)	–	0.6 (0.1–3.9)	1.4 (0.5–3.4)	3.3 (1.7–6.3)	1.4 (0.5–3.8)	0.8 (0.1–5.3)	1.2 (0.3–4.6)	–	7.3 (1.1–41.6)
Breast	Campoy et al, 2025 [14]	Retrospective, not corrected for bias (19)	–	–	17.9 (12.2–25.5)	–	11.1 (5.3–22.1)	–	31.1 (21.9–40.7)	–	37.7 (27.5–47.8)
		Population–based (1, UK Biobank)	–	–	10.1 (3.2–29.9)	–	6.8 (1.0–39.1)	–	8.3 (4.4–15.2)	–	9.2 (5.8–14.3)
		Prospective (1, PLSD)	–	–	10.5 (7.4–15.1)	–	12.6 (8.9–17.8)	–	11.1 (5.8–31.5)	–	8.1 (2.8–46.5)
Prostate	Campoy et al, 2025 [14]	Prospective (1, PLSD)	–	7.6 (4.8–11.8)	–	16.4 (12.0–22.1)	–	5.8 (2.8–11.8)	–	3.3 (0.5–21.5)	–
Sarcoma	Dominguez-Valentin et al, 2020 [44]	Prospective (1, PLSD)	–	0.73 (0–1.56)		4.24 (0.54–7.93)		0 (0–0)		1.57 (0–4.63)	

*LS* Lynch Syndrome, *PLSD* Prospective LS Database

**Table 8:** Relative other cancer risks in LS from population- and laboratory-based cohorts. Reported as odds ratios, relative risks or standardized incidence ratios (95% confidence interval)

Cancer Type	Study, year	All LS Genes		*MLH1*	*MSH2*	*MSH6*	*PMS2*
		M	F				
Ovary	Drogan et al. 2024* [26]	–		2.1 (1.4–3.2)	4.1 (3.2–5.3)	2.2 (1.8–2.7)	1.2 (1.0–1.5)
Kidney	Nassour et al. 2023*** [37]	7.7 (3.7–15.1)		–	–	–	–
	Drogan et al. 2024* [26]	–		1.0 (0.5–2.3)	2.8 (1.8–4.3)	0.8 (0.4–1.45)	0.8 (0.4–1.5)
Bladder	Nassour et al. 2023*** [37]	4.0 (1.2–12.8)		–	–	–	–
Breast	Rosenblum et al., 2020* [18]	–	2.5 (0.9–6.9)	–	–	–	–
	Stoll et al. 2020^Ŧ^ [41]	–		0.5 (0.4–0.7)	0.50 (0.4–0.6)	0.7 (0.6–0.8)	0.8 (0.7–0.9)
	Drogan et al. 2024*[26]	–		1.3 (1–1.7)	1.1 (0.9–1.4)	1.0 (0.9–1.2)	0.9 (0.8–1.0)
Non CRC/EC LS cancers#	Park et al., 2025* [16]	2.7 (2.0–3.6)		–	–	–	–

*LS* Lynch Syndrome

^*^Odds ratio; ***Relative risk; ^Ŧ^Standardized incidence ratio

^#^Non-CRC/EC cancers include stomach, small bowel, ovaries, hepatobiliary tract, urothelial, prostate and pancreas

*Ovarian Cancer*: The most common histological subtypes of ovarian cancer in LS are mixed type, clear cell carcinoma and endometrioid carcinoma [36], though most studies do not distinguish by subtype. In the meta-analysis by Campoy et al., the risk of ovarian cancer was highest for *MLH1* and *MSH2* PV carriers, though was relatively uncommon before age 50 [14]. There was limited data to be able to provide robust risk estimates for ovarian cancer in *MSH6/PMS2* PV carriers due to a lack of retrospective studies that corrected for ascertainment bias included in the meta-analysis that estimated penetrance of ovarian cancer by age 70 in these genes. In PLSD, ovarian cancer risks were increased in *MSH2* and *MLH1* PV carriers (13.4% and 8%, respectively) as well as *MSH6* PV carriers (6.3%) [19].

A meta-analysis by Karimi et al. calculated the prevalence of ovarian cancer in female LS carriers to be 5.7% [29]. This was highest in *MSH2* (6.1%) and *MSH6* (5%), followed by *MLH1* (2.5%) and *PMS2* (2%). *MSH2* carriers had the highest risk of ovarian cancer compared to the other LS genes, though the confidence interval spanned 1.0 (1.35, CI 0.90–2.08).

Relative ovarian cancer risk was highest in *MSH2* in the laboratory-based cohort but was also significant for *MLH1* and *MSH6* carriers [26]. Only 1.29% of women undergoing testing for ovarian cancer had LS in this study. Ovarian cancer was only reported in one female with LS in the Bio*Me* study and was not reported in the All of Us study, so therefore risks of ovarian cancer were not estimated for population-based studies [16, 18].

*Urological Cancer*: Urological tract cancers, such as kidney, renal pelvis, urothelial and bladder cancer, may be reported as individual or organ-system cancer risk. These data should be interpreted cautiously, as the tissue of origin may have been misreported. In the Campoy et al. meta-analysis, cumulative lifetime risks of cancers of the urogenital tract were elevated, primarily for *MSH2* PV carriers [14]. Lifetime risks of urothelial cancers could not be calculated in *PMS2* PV carriers due to a lack of robust data, and risk of bladder cancer was not able to be estimated for all genes in retrospective studies corrected for ascertainment bias.

Another meta-analysis examining the risks of urinary tract cancers in confirmed LS PV carriers showed that individuals with LS had a higher risk of both bladder (RR 7.48 [3.70, 15.13]) and kidney cancers (RR 3.97 [1.23, 12.81]) [37]. However, these data were not stratified by sex or affected gene, which other studies have shown these factors affect risks for these cancers [14, 28, 32]. In a retrospective study, bladder and other urothelial cancers were more common in males (cumulative risk by age 70: 5.5 95% CI 2.7–8.3 and 9.4 95% CI 4.6–14.1, respectively) than females (cumulative risk by age 70: 3.5 95% CI 1.4–5.5 and 6.0 95% CI 2.9–9.1, respectively) [32].

Risks of urological cancers were not estimated in the population-based studies, as no participants with LS had a personal history of urothelial cancer [16, 18]. In the laboratory-based cohort, only *MSH2* PVs were associated with an increased risk of kidney cancer [26]. LS was found in 2.39% of people with kidney cancer undergoing genetic testing. Risks of other urological cancers could not be estimated due to low power.

*Brain:* Brain cancer was identified in 41/288 (14%) LS families (diagnosis age ranging from 2 to 73 years) enrolled in a Danish prospective registry and were retrospectively reviewed [38]. 68% of the patients with brain tumors had a PV in *MSH2*, 15% had either a *MLH1* or *MSH6* PV, and only 2% were in *PMS2* PV carriers. The most common subtype was glioblastoma (56%), followed by astrocytoma (22%) and oligodendroglioma (9%). Two siblings with constitutional mismatch repair deficiency (biallelic *MSH6* PVs) and one other child with one *MSH6* PV and one *MSH6* variant of uncertain significance were included in these estimates and therefore may inflate the risks of brain cancer for *MSH6* heterozygotes. Cumulative risks of brain cancer (all subtypes) were 0.5 (95% CI 0.0–0.87) in *MLH1*, 2.5 (95% CI 1.66–3.36) in *MSH2* and 0.8 (95% CI 0.17–2.34) for *MSH6* PV carriers [38].

Risks of brain cancer could not be calculated in the laboratory-based cohort, in population-based studies or in the retrospective studies corrected for ascertainment bias in the meta-analysis by Campoy et al. due to low power [14, 16, 18, 26]. Similar to the referenced Dutch study [38], in the meta-analysis, risk of brain tumors in retrospective studies not corrected for bias showed the highest risk of brain cancer in *MSH2* carriers [14]. In the prospective studies, the risk was highest for female *PMS2* carriers and male *MSH2* carriers, though the confidence interval is wide [14].

*Skin:* Benign and malignant skin neoplasms have been reported in LS. Presence of sebaceous neoplasms in LS is sometimes referred to as Muir-Torre syndrome.

A meta-analysis of nine studies of varying study design included 1569 patients with sebaceous neoplasms found that 0.8–29.0% of patients with sebaceous adenomas and 6.6% of patients with sebaceous carcinomas had LS [39]. Males with LS were more likely to have sebaceous neoplasms than female LS carriers, and younger patients with sebaceous neoplasms were more likely to have LS [39].

A Dutch retrospective study, not included in the previously mentioned meta-analysis, reported skin neoplasms in 8.2% of LS PV carriers [40]. Risks of squamous cell carcinoma and sebaceous adenomas were increased in patients with LS compared to the general population (7.8 ± 2.6%), and cumulative risk by age 70 of any malignant skin tumor was 21.1% (± 4.5%). Skin neoplasms were more likely to occur in individuals with PVs in *MLH1/MSH2* compared to *MSH6/PMS2* (HR 4.21 [1.68, 10.5]).

Risks of skin neoplasms could not be calculated in the population-based studies, laboratory-based cohort or in the retrospective studies corrected for ascertainment bias in the meta-analysis by Campoy et al. due to low power [14, 16, 18, 26].

## Breast and prostate cancer

It remains debated whether breast and prostate cancer risks are increased in LS patients. Breast and prostate are the most common cancers in women and men, respectively which makes it more challenging to determine elevated risks in LS. Tables 7 and 8 summarizes current breast and prostate risk estimates.

*Breast:* The meta-analysis by Karimi et al. calculated the prevalence of breast cancer to be 11%, which is similar to the prevalence of breast cancer in the general population [29]. Risks were highest in the genes typically considered lower penetrance (13% in *MSH6* and 18% in *PMS2*) compared to higher penetrance genes (4% in *MLH1* and 8% in *MSH2*). Cumulative risk of breast cancer in the retrospective studies corrected for ascertainment bias was not calculated in the Campoy et al. meta-analysis, though in prospective studies was 8–12%, which is similar to lifetime breast cancer risk in the general population [14].

In a laboratory-based cohort, 0.5% (891/173,945) women with breast cancer had LS, most commonly in *PMS2* (0.3%) [26]. None of the LS genes were significantly associated with an increased risk of breast cancer. A previous study published in collaboration with the same genetic testing laboratory also reported no increased risk of breast cancer in LS, though reported standardized incidence ratios, rather than odds ratios [41]. In this study, most *MLH1* and *MSH2/EPCAM* PV carriers were clinically ascertained for suspicion of LS (53%), while most *MSH6* and *PMS2* carriers were ascertained for suspicion of Hereditary Breast and Ovarian Cancer (79.2%), indicating that these may be incidental findings.

*Prostate:* Prostate cancer, like breast cancer, has been hypothesized to be related to LS. A meta-analysis of 23 studies suggests that the risk of prostate cancer may be 2–3-fold increase compared to the general population, though almost all studies reported on men of European descent, so may not be applicable for all ethnicities, and did not delineate risk by mutated gene [42]. Some studies included in this meta-analysis did have molecular data, reporting microsatellite instability and loss of *MSH2/MSH6* on immunohistochemical staining, suggesting LS PV as the underlying cause of the prostate cancer. Cumulative risk of prostate cancer in the retrospective studies corrected for ascertainment bias was not calculated in the Campoy et al. meta-analysis [14].

In a study of 235 males with LS, cumulative incidence of prostate cancer was estimated to be 38% (95% CI 27–50%) [3]. Cumulative incidence of clinically significant prostate cancer, as well as overall prostate cancer risks, were reported. Men with *MSH2/EPCAM* PVs had higher risks compared to *MLH1* and *MSH6* in the overall cohort and in the clinically significant prostate cancer cohort. Family history of prostate cancer increased the risk (OR 3.00 95% CI 1.35–6.71). Interestingly, none of the prostate cancers diagnosed in males with *MLH1* or *PMS2* PVs demonstrated abnormal immunohistochemistry, indicating that these may be sporadic cancers.

Relative risk of prostate cancer could not be estimated in the laboratory-based cohort or in the population-based studies due to low power [16, 18, 26].

## Other cancers

Mismatch repair deficiency due to germline LS PV has been reported in a variety of non-traditional LS spectrum cancer types, suggesting that all individuals with mismatch repair deficient tumors regardless of origin should be offered germline LS testing [43]. This may continue to expand the phenotype associated with LS.

In a retrospective study, sarcoma (SIR 2.13 95% CI 0.58–5.46), lung (SIR 1.98 95% CI 0.88–4.94), melanoma (SIR 0.51 0.10–1.48), prostate (SIR 1.16 95% CI 0.43–2.52), and breast cancer (SIR 1.63 95% CI 0.98–2.28) were not associated with LS; however, all genes were grouped together so do not demonstrate individual gene risk or risk by sex at birth [33]. This study also did not find a correlation between LS and bladder cancer (SIR 1.03 95% CI 0.41–2.11), which is in contrast to some other studies [32, 37]. Interestingly, thyroid cancer risk was elevated (SIR 2.81 95% CI 1.45–4.90), which warrants further investigation [33]. A prospective study reported 14 cases of sarcoma, with the highest risk in *MSH2* PV carriers (4.24% 95% CI 0.54–7.93 cumulative incidence at 75 years), which is significantly higher than sarcoma risk in the general population [44].

## Conclusions

Estimating accurate cancer risks for LS remains challenging, particularly for lower penetrance genes and rarer LS cancers. Variability in study design requires caution when interpreting results and comparing studies to one another. Cumulative incidence remains the clearest metric for patient communication.

Population-based studies highlight that LS is widely underdiagnosed, partly because as many as 80% of cases are due to PVs in *MSH6* and *PMS2* which have lower penetrance and later age of cancer onset compared to *MLH1* and *MSH2*. *MLH1* and *MSH2* consistently exhibit higher cancer risks, except for endometrial cancer, in which risks for *MSH6* carriers is comparable. Males may have a higher cumulative risk of colorectal cancer compared to females, though current guidelines do not reflect this sex difference. *MSH2* PV carriers may be more predisposed to non-colorectal and non-endometrial cancers compared to other genes. Risk estimates for non-colorectal cancer in *EPCAM* PV carriers are limited, even in large cohorts. Cancer risks from these various studies support current cancer screening recommendations and the notion that LS is five distinct cancer predisposition syndromes.

To further refine estimates and improve management recommendations, large, prospective longitudinal studies that minimize bias, prioritize complete data collection, utilize verified personal and family history and include lower penetrance PV carriers are needed. Providing accurate risk estimates for non-colorectal and non-endometrial cancers and older individuals may inform screening recommendations. To date, PLSD has been the primary prospective data source for LS cancer risk estimates. Additional prospective consortia, such as the North American LINEAGE consortium [45], will provide risk estimates from additional regions of the world.

As advances in variant-specific cancer risk, polygenic risk scores and environmental modifiers are integrated into risk assessment, estimation of cancer risks will increase in complexity. However, this will allow health care professionals to give LS patients and their family members highly personalized cancer risks and surveillance recommendations in future.

## Data Availability

No datasets were generated or analysed during the current study.
